# A pilot study to search possible mechanisms of ultralong gonadotropin-releasing hormone agonist therapy in IVF-ET patients with endometriosis

**DOI:** 10.1186/s13048-014-0100-8

**Published:** 2014-10-21

**Authors:** Hiroshi Tamura, Akihisa Takasaki, Yasuhiko Nakamura, Fumitaka Numa, Norihiro Sugino

**Affiliations:** Department of Obstetrics and Gynecology, Yamaguchi University Graduate School of Medicine, Minamikogushi 1-1-1, Ube, 755-8505 Japan; Department of Obstetrics and Gynecology, Saiseikai Shimonoseki General Hospital, Yasuokacho 8-5-1, Shimonoseki, 759-6603 Japan; Department of Obstetrics and Gynecology, Yamaguchi Grand Medical Center, Oazaosaki 77, Foufu, 747-8511 Japan; Department of Obstetrics and Gynecology, Tokuyama Central Hospital, Koudacho 1-1, Syunan, 745-8522 Japan

**Keywords:** Endometriosis, GnRH agonist, Cytokine, Oxidative stress, Melatonin

## Abstract

**Background:**

Additional treatment with a gonadotropin-releasing hormone (GnRH) agonist (GnRHa) before IVF-ET (ultralong GnRHa therapy) has been reported to improve the outcome of IVF-ET in endometriosis patients. However, the mechanism of ultralong GnRHa therapy is unclear. It is suggested that inflammatory cytokines and oxidative stress contribute to infertility in endometriosis patients. Therefore, in order to search a possible mechanism of ultralong GnRHa therapy, we investigated the effect of ultralong GnRHa therapy on intrafollicular concentrations of tumor necrosis factor alpha (TNFα), oxidative stress markers, and antioxidants in patients with endometriosis.

**Methods:**

Twenty-three infertile women with Stage III or IV endometriosis were recruited for this study. Eleven patients received three courses of GnRHa (1.8 mg s.c. every 28 days), followed by a standard controlled ovarian hyperstimulation (COH) for IVF-ET (ultralong group). The other 12 patients received a standard COH with mid-luteal phase GnRHa down-regulation (control group). The numbers of matured follicles and retrieved oocytes, fertilization rates, implantation rates, clinical pregnancy rate, and intrafollicular concentrations of TNFα, 8-hydroxy-2’-deoxyguanosine (8-OHdG) and hexanoyl-lysine adduct (HEL) as oxidative stress markers, and melatonin and Cu,Zu-superoxide dismutase (Cu,Zn-SOD) as antioxidants were compared between the two groups.

**Results:**

The numbers of mature follicles and retrieved oocytes, and fertilization rates did not differ between the two groups. Implantation rates and pregnancy rates tended to be higher in the ultralong group (21.4% and 27.3%, respectively) compared with the control group (8.3% and 8.3%, respectively). TNFα concentrations in the follicular fluid were significantly lower in the ultralong group (5.8 ± 3.2 pg/ml) than those in the control group (10.6 ± 3.2 pg/ml). Follicular concentrations of 8-OHdG concentrations were significantly lower in the ultralong group (5.7 ± 1.6 ng/ml) than those in the control group (6.6 ± 1.5 ng/ml), while melatonin concentrations were significantly higher in the ultralong group (139 ± 46 pg/ml) compared with the control group (86 ± 27 pg/ml).

**Conclusions:**

Ultralong GnRHa therapy reduces the detrimental effects of cytotoxic cytokines and oxidative stress in the ovary in patients with endometriosis.

## Background

Endometriosis is considered to be the most intractable cause of female infertility [1,2]. Possible causes of the infertility include poor quality of oocytes and embryos [3], impaired fertilization [4,5], and impaired implantation [6], each of which can be induced by inflammation and oxidative stress in the pelvic cavity [7-9]. In fact, increased levels of inflammatory cytokines such as interleukin-6 (IL-6), interleukin-8 (IL-8), and tumor necrosis factor-alpha (TNFα) have been observed in the peritoneal fluid in patients with endometriosis [10]. TNFα, which is produced by activated macrophages and NK cells, is a key molecule in endometriosis. TNFα in the peritoneal cavity impairs oocytes and embryos by its cytotoxicity [11,12]. TNFα also increases prostaglandin production by endometrial epithelial cells, which in turn initiates a surge of other inflammatory cytokines in the endometrium [13,14]. This surge of inflammatory cytokines has been proposed as a cause of endometriosis-related implantation failure [13,14]. In addition, endometriosis patients showed high concentrations of TNFα in ovarian follicular fluids, which is associated with poor oocyte quality or impaired fertilization [11].

Chronic inflammation in the pelvic cavity of endometriosis patients also induces oxidative stress in both the pelvic cavity and reproductive organs. Increased and activated macrophages and polymorphonuclear leucocytes in the peritoneal fluid produce large amounts of reactive oxygen species (ROS) in patients with endometriosis [15]. Oxidative stress levels in the ovarian follicular fluid are higher in patients with endometriosis than in other infertility patients without endometriosis [16]. ROS production by endometrium is increased in endometriosis patients [17]. Oxidative stress causes detrimental effects on cells through lipid peroxidation, protein oxidation, and DNA damage. Oxidative stress in the peritoneal cavity is one of the causes of endometriosis-associated infertility [8]. Oxidative stress reduces oocyte quality [18-20] and impairs endometrial receptivity [21]. Together, these findings strongly suggest that oxidative stress contributes to infertility in endometriosis patients.

The pregnancy outcomes of endometriosis patients who undergo in vitro fertilization and embryo transfer (IVF-ET) are generally poor [22], and there are no effective treatments for endometriosis-related infertility. Additional treatment with a gonadotropin-releasing hormone (GnRH) agonist (GnRHa) before IVF-ET (ultralong GnRHa therapy) improved the outcome of IVF-ET in endometriosis patients, as shown by increased numbers of retrieved oocytes and transferred embryos, and higher implantation and pregnancy rates [23-25]. However, the mechanism of ultralong GnRHa therapy is unclear. Therefore, we investigated the concentrations of TNFα and oxidative stress in ovarian follicular fluids to see if they can shed light on the mechanism by which ultralong GnRHa therapy improves the IVF-ET outcome in endometriosis patients.

## Materials and methods

### Patients and clinical study

This study was reviewed and approved by the Institutional Review Board of Yamaguchi University Graduate School of Medicine. Informed consent was obtained from all the patients in this study. Twenty-three infertile female patients with endometriosis were recruited for this study. The mean age ± S.D. of the patients was 34.0 ± 3.3 yr. with a range of 28–40 yr. Diagnosis of endometriosis was confirmed by laparoscopy, laparotomy, or transvaginal aspiration of the ovarian endometrial cyst. Severity of endometriosis was scored according to the four-stage classification of the revised American Society for Reproductive Medicine (rASRM) score, and all patients had stage III or IV endometriosis. Patients were nonsmokers and free from major medical illness including hypertension; all were interested in becoming pregnant. Patients were excluded if they had myoma, adenomyosis, a congenital uterine anomaly, or if they used any kind of sex-steroidal agent including estrogens, progesterone, androgens, and OC.

Eleven patients received three courses of GnRHa (1.8 mg s.c. every 28 days) (buserelin acetate, Suprecur; Mochida Pharmaceutical Co. Ltd., Tokyo, Japan), followed by a standard controlled ovarian hyperstimulation (COH) for IVF-ET (ultralong group). Withdrawal bleeding was induced using estrogen (Premarin: conjugated estrogens tablets, Pfizer Pharmaceutical Co. Ltd., Japan) and progesterone (Duphaston: dydrogesterone tablets, Daiichi-Sankyo Co. Ltd., Japan) before COH. COH was initiated from the 2nd day of the IVF-ET cycle by injection of 225 IU FSH (Folyrmon P; Fuji Pharmaceutical Co. Ltd., Tokyo, Japan) for 3 days, followed by a daily injection of 150 IU HMG (HMG F; Fuji Pharmaceutical Co. Ltd., Tokyo, Japan). Nasal spray GnRHa (900 μg/day buserelin acetate, Suprecur; Mochida Pharmaceutical) was also given from the 2nd day of the IVF-ET cycle to continuously suppress pituitary gonadotropin secretion until the injection of HCG (HCG Mochida 10,000 IU; Mochida Pharmaceutical) for ovulation induction. Ultralong group included one case with a male factor.

Twelve patients received a standard COH with mid-luteal phase GnRHa down-regulation (control group). In the control group, nasal spray GnRHa (900 μg/day) was given from the mid-luteal phase in the previous cycle to the time of HCG injection for ovulation induction of the IVF-ET cycle. COH was given in a manner similar to the ultralong group described above.

When leading follicles reached 18 mm or more, HCG was injected for ovulation induction. Oocyte retrieval was carried out 35 h after HCG injection. Each mature follicle (more than 18 mm in diameter) was aspirated separately and the follicular fluid containing the oocyte was collected. Immediately after removal of the oocyte, each of the follicular fluids was centrifuged at 300 x g for 15 min to remove cellular components. The supernatant from each follicle was mixed in each patient and was kept at –80C until assayed. The numbers of matured follicles, retrieved oocytes and fertilized oocytes, and fertilization rates, implantation rates, and clinical pregnancy rates were compared between the two groups. Concentrations of TNFα, IL-6, and oxidative stress markers; 8-hydroxy-2’-deoxyguanosine (8-OHdG) as a marker of DNA damage and hexanoyl-lysine adduct (HEL) as a marker of lipid peroxidation, and Cu,Zu-superoxide dismutase (Cu,Zn-SOD) and melatonin, as antioxidants, in follicular fluids were measured using an ELISA kit or a radioimmunoassay described below.

### Measurement of TNFα, IL-6, oxidative stress markers, and antioxidants in follicular fluids

Concentrations of TNFα and IL-6 were measured using a Human TNFα ELISA kit and Human IL-6 ELISA kit (Thermo Fisher Scientific Pierce Biotechnology, Rockford, USA), respectively. Each sample of follicular fluid (50 μl) was used for duplicate assay according to the assay protocol. The sensitivity of TNFα was 2 pg/ml, and the coefficients of variation (CV) for intra- and inter-assay were 4.5% and 5.2%, respectively. The sensitivity of IL-6 was 1 pg/ml, and the CV for intra- and inter-assay were <10%.

8-OHdG concentrations were measured using a New 8-OHdG Check ELISA (Japan Institute for the Control of Aging, Nikken SEIL Co. Ltd., Shizuoka, Japan) as we reported previously [26,27]. Each sample of follicular fluid (50 μl) filtered using an ultrafilter (cut off molecular weight 10 kDa) was used for duplicate assay. The sensitivity of 8-OHdG was 0.5 ng/ml, and the CV for intra- and inter-assay were 5.5% and 6.1%, respectively.

HEL concentrations were measured using an ELISA kit (Japan Institute for the Control of Aging) as we reported previously [26,27]. Each sample of follicular fluid (50 μl) was pretreated with chymotrypsin to perform proteolysis, and filtered using an ultrafilter (cut off molecular weight 10 kDa) for duplicate assay. The minimal detectable concentration of HEL was estimated to be 2 nmol/L.

Cu,Zn-SOD concentrations were measured using a Human Cu/Zn-superoxide dismutase ELISA kit (Northwest Life Science Specialties, LLC, USA) as we reported previously [26,27]. Each sample of follicular fluid (20 μl) was used for duplicate assay according to assay protocol. The sensitivity of Cu,Zn-SOD was 0.04 ng/ml, and the CV for intra- and inter-assay were 5.1% and 5.8%, respectively.

Intrafollicular concentrations of melatonin were measured by radioimmunoassay (RIA) as we reported previously [28]. Each sample of follicular fluid (500 μl) was used for duplicate assay. The sensitivity of the assay was 4.2 pg/ml, and the CV for intra- and inter-assay were 6.3% and 4.9%, respectively.

### Statistical analysis

Statistical analysis was carried out with SPSS for Windows 13.0. The Mann–Whitney U-test using the Bonferroni correction and Fisher’s test were employed as appropriate. Correlations were analyzed using Spearman’s rank correlation coefficient. Differences were considered to be significant if *P* <0.05.

## Results

There was no significant difference in the mean age of the patients between the two groups (Table 1). These treatments resulted in the ultralong group receiving a greater dose of gonadotropin and a longer duration of ovarian stimulation (Table 1). The numbers of mature follicles and retrieved oocytes, and fertilization rates were not significantly different between the two groups (Table 1). Embryo transfer was carried out in 8 of 12 cases in the control group and in 8 of 11 cases in the ultralong group (Table 1). The implantation rate and pregnancy rate were higher in the ultralong group (21.4% and 27.3%, respectively) compared with the control group (8.3% and 8.3%, respectively), but the differences were not significant (Table 1).

**Table 1:** **Clinical characteristics and IVF-ET data**

	**Control group**	**Ultralong group**	***P*** **value**
No. of patients	12	11	
Age (yrs)	34.5 ± 3.4	33.5 ± 3.3	0.45
Gonadotropin dose (IU)	1502 ± 377	2209 ± 849^a^	0.037
Duration of COH (days)	8.6 ± 1.9	11.5 ± 3.4^a^	0.032
Estradiol (pg/ml)	1995 ± 1054	1280 ± 892	0.098
No. of follicles (≧15 mm)	7.1 ± 2.7	6.6 ± 3.8	0.79
No. of mature follicles (≧18 mm)	3.0 ± 2.0	3.2 ± 2.8	0.79
No. of oocytes retrieved	5.0 ± 2.9	5.7 ± 4.1	0.70
Fertilization rate (%)	51.7 (31/60)	39.7 (25/63)	0.21
Cases of IVF/ICSI	11 / 1	8 / 3	
Cases of embryo transfer	8	8	0.79
No. of embryos transferred	1.2 ± 0.8	1.3 ± 1.0	0.74
Cases of 2–4 cell embryo/blastcyst (cryopreserved)	4 (1) / 4 (1)	6 (0) / 2 (0)	
Implantation rate (%)	8.3 (1/12)	21.4 (3/14)	0.35
Pregnancy rate (%)	8.3 (1/12)	27.3 (3/11)	0.23

Twenty-three infertile women with Stage III or IV endometriosis were recruited for this study. Eleven patients received three courses of GnRHa (1.8 mg s.c. every 28 days), followed by a standard controlled ovarian hyperstimulation (COH) for IVF-ET (ultralong group). Twelve patients received a standard COH with mid-luteal phase GnRHa down-regulation (control group). Data are shown as the mean ± SD. a; significant difference (Fisher’s test or the Mann–Whitney U-test using the Bonferroni correction).

**Figure 1:**
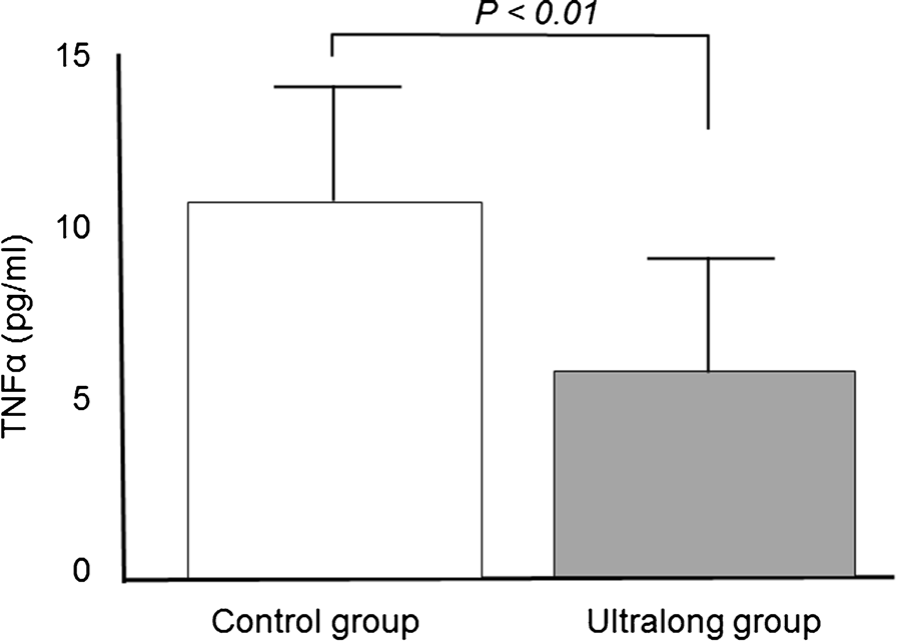
**Tumor necrosis factor alpha (TNFα) concentrations in follicular fluids.** Twenty-three infertile women with Stage III or IV endometriosis were recruited for this study. Eleven patients received three courses of GnRHa (1.8 mg s.c. every 28 days), followed by a standard controlled ovarian hyperstimulation (COH) for IVF-ET (ultralong group). Twelve patients received a standard COH with mid-luteal phase GnRHa down-regulation (control group). TNFα concentrations were measured in the follicular fluid obtained at the time of oocyte retrieval. Values are mean ± SD. Statistical analysis was employed with the Mann–Whitney U-test using the Bonferroni correction.

**Figure 2:**
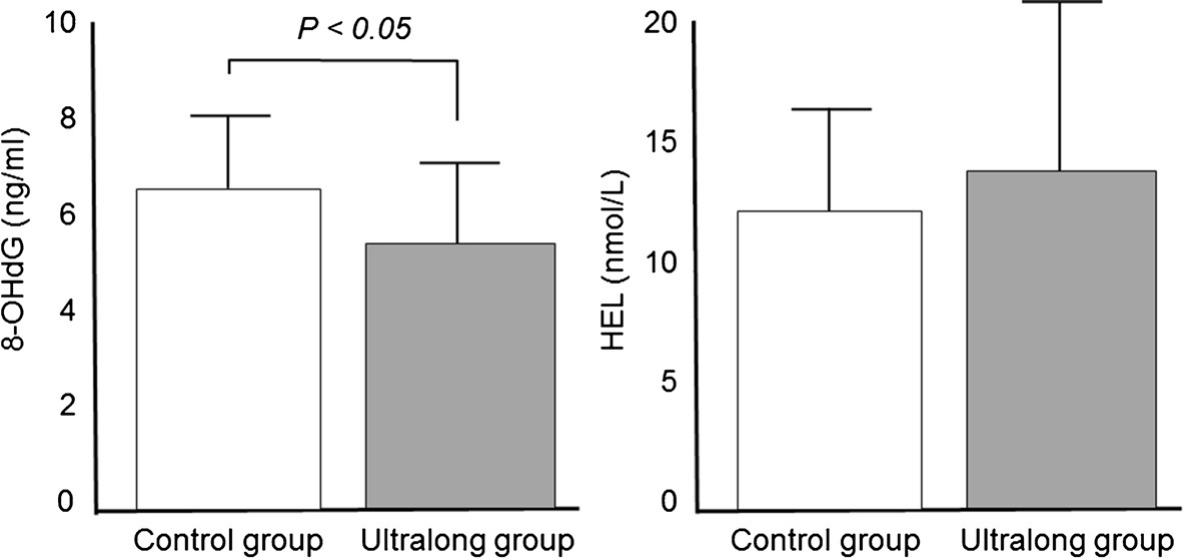
**Concentrations of oxidative stress markers in follicular fluids.** Twenty-three infertile women with Stage III or IV endometriosis were recruited for this study. Eleven patients received three courses of GnRHa (1.8 mg s.c. every 28 days), followed by a standard controlled ovarian hyperstimulation (COH) for IVF-ET (ultralong group). Twelve patients received a standard COH with mid-luteal phase GnRHa down-regulation (control group). The levels of oxidative stress markers; 8-hydroxy-2’-deoxyguanosine (8-OHdG) as a marker of DNA damage and hexanoyl-lysine adduct (HEL) as a marker of lipid peroxidation, were measured in the follicular fluid obtained at the time of oocyte retrieval. Values are mean ± SD. Statistical analysis was employed with the Mann–Whitney U-test using the Bonferroni correction.

**Figure 3:**
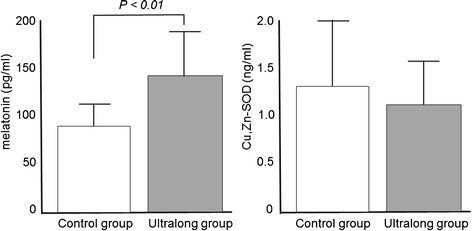
**Concentrations of antioxidants in follicular fluids.** Twenty-three infertile women with Stage III or IV endometriosis were recruited for this study. Eleven patients received three courses of GnRHa (1.8 mg s.c. every 28 days), followed by a standard controlled ovarian hyperstimulation (COH) for IVF-ET (ultralong group). Twelve patients received a standard COH with mid-luteal phase GnRHa down-regulation (control group). The levels of Cu,Zu-superoxide dismutase (Cu,Zn-SOD) and melatonin, as antioxidants, were measured in the follicular fluid obtained at the time of oocyte retrieval. Values are mean ± SD. Statistical analysis was employed with the Mann–Whitney U-test using the Bonferroni correction.

TNFα concentrations in the follicular fluid were significantly lower in the ultralong group (5.8 ± 3.2 pg/ml) than in the control group (10.6 ± 3.2 pg/ml) (Figure 1). IL-6 was not detected in the follicular fluid in either group. 8-OHdG concentrations were slightly but significantly lower in the ultralong group (5.7 ± 1.6 ng/ml) than in the control group (6.6 ± 1.5 ng/ml), whereas the follicular HEL concentrations were not significantly different (Figure 2). Melatonin concentrations were significantly higher in the ultralong group (139.2 ± 45.7 pg/ml) than in the control group (85.6 ± 27.4 pg/ml), while Cu,Zn-SOD concentrations were not significantly different between the two groups (Figure 3).

The correlations between TNFα, 8-OHdG and melatonin concentrations in the follicle were analyzed using Spearman’s rank correlation coefficient. There were slight positive correlations between TNFα and 8-OHdG (R = 0.1178, P = 0.5924), and negative correlations between TNFα and melatonin (R = −0.0893, P = 0.6852) and melatonin and 8-OHdG (R = −0.3976, P = 0.0602). However, the trends were not statistically significant because of a small sample size.

## Discussion

The present result clearly showed that the concentrations of a cytotoxic cytokine (TNFα) and oxidative stress (8-OHdG) in follicular fluids were significantly lower in the ultralong GnRHa therapy group than in the control group, suggesting a potential mechanism that additional GnRHa treatment before IVF-ET improves the pregnancy outcome of IVF-ET by reducing the detrimental effects of cytotoxic cytokines and oxidative stress in the peritoneal environment or implantation environment in patients with endometriosis. TNFα and oxidative stress in ovarian follicles not only damage oocytes and embryos leading to the impaired fertilization, but also impair endometrial receptivity leading to implantation failure in patients with endometriosis [10-14].

Although ultralong GnRHa therapy reduced the concentrations of TNFα and oxidative stress markers in ovarian follicles, it is unclear whether it also reduced them in the peritoneal cavity and in the endometrium. Since it is reported that the cytokine levels were decreased by GnRHa treatment in the peritoneal cavity as well as in the ovary [29,30], there is a possibility that the hostile environment of the peritoneal cavity and endometrial cavity was improved by ultralong GnRHa therapy in this study. Therefore, we hypothesize that the decrease in TNFα and oxidative stress by ultralong GnRHa therapy may have contributed to the improvement of implantation rate and pregnancy rate.

Interestingly, ultralong GnRHa therapy increased the melatonin concentrations in the follicular fluid (Figure 3). Melatonin is a hormone secreted by the pineal gland, and regulates a variety of central and peripheral actions related to circadian rhythms and reproduction. Melatonin is a powerful free radical scavenger and a broad-spectrum antioxidant [31,32]. We previously demonstrated that melatonin is present in human ovarian follicles and that its concentration increases during follicular growth [18-20]. We also reported that melatonin is taken up into the follicular fluid from the blood, and that it protects oocytes from ROS within the follicle during ovulation [18-20,33]. Reduced oxidative stress and increased antioxidant activities by melatonin in follicular fluids by ultralong GnRHa therapy may also have contributed to the improvement of implantation rate and pregnancy rate. The mechanism by which ultralong GnRHa therapy increases the melatonin concentration in the follicle is unclear. We speculate that ultralong GnRHa therapy may have improved the function of the follicle by reducing inflammation of the ovary so that the follicle can effectively take up melatonin.

It is unclear how TNFα, oxidative stress, and melatonin interacts each other. There were slight positive correlations between TNFα and 8-OHdG, and negative correlations between TNFα and melatonin, and melatonin and 8-OHdG, although the trends were not statistically significant. These results may suggest that TNFα induces oxidative stress and decreases melatonin levels in the follicle. In other words, the reduced TNFα by ultralong GnRHa therapy may be responsible for the decrease in oxidative stress and the increase in melatonin in the follicle.

On the other hand, as a demerit of the ultralong GnRHa therapy, our results showed a need for greater gonadotropin doses and longer COH days than in patients who received a standard mid-luteal GnRHa down-regulation protocol, which is also consistent with previous reports [34].

Unfortunately, the present study did not clearly show that ultralong GnRHa therapy improves the fertility of patients with endometriosis. This may be due to the small sample size of this study. A large-scale randomized controlled trial will be necessary to evaluate the efficacy of ultralong GnRHa therapy on pregnancy outcome in patients with endometriosis. It is also interesting to investigate whether there are any differences in follicular levels of TNFα, 8-OHdG, and melatonin between the follicles containing fertilized and unfertilized, implanted and non-implanted, or pregnant and non-pregnant oocytes.

## Conclusions

This study suggested a possible mechanism of ultralong GnRHa therapy to improve the pregnancy outcome of IVF-ET, which is the reduction of the detrimental effect of cytokines and oxidative stress in the peritoneal environment or implantation environment in patients with endometriosis.
